# The impact of strict measures as a result of the COVID-19 pandemic on the spatial pattern of the demand for police: case study Antwerp (Belgium)

**DOI:** 10.1186/s40163-021-00156-7

**Published:** 2021-09-27

**Authors:** Maite Dewinter, Christophe Vandeviver, Philipp M. Dau, Tom Vander Beken, Frank Witlox

**Affiliations:** 1 Department of Geography, Ghent University, Krijgslaan 281, 9000 Ghent, Belgium; 2 Department of Criminology, Criminal Law and Social Law, Ghent University, Universiteitstraat 4, 9000 Ghent, Belgium; 3 Research Foundation-Flanders (FWO), Egmontstraat 5, 1000 Brussels, Belgium; 4 Department of Geography, University of Tartu, Vanemuise 46, 51014 Tartu, Estonia; 5 College of Civil Aviation, Nanjing University of Aeronautics and Astronautics, Nanjing, 210016 China

**Keywords:** Spatial point pattern test, Police demand, Lockdown, Street segments, Emergency call data

## Abstract

COVID-19 impacts the daily lives of millions of people. This radical change in our daily activities affected many aspects of life, but acted as well as a natural experiment for research into the spatial distribution of 911 calls. We analyse the impact of the COVID-19 measures on the spatial pattern of police interventions. Crime is not uniformly distributed across street segments, but how does COVID-19 affect these spatial patterns? To this end, Gini coefficients are calculated and a proportion differences spatial point pattern test is applied to compare the similarity of the patterns of incidents before, during, and after the first lockdown in Antwerp, Belgium. With only essential mobility being allowed, the emergency call pattern has not significantly changed before, during or after this lockdown, however, a qualitative shift in police officer’s daily work may have had an effect on the daily operation of the Antwerp police force.

## Introduction

The spread of the COVID-19 virus has resulted in unprecedented measures restricting travel and activity participation, which has had a huge impact on our hyper-mobile society (Musselwhite et al., 2020; van Wee & Witlox, 2021). In many countries and particularly at city level, social distancing, i.e., reducing interactions between individuals in order to slow down the spread of the virus, has become the new norm. Public authorities in general, and police in particular face the vast societal challenge of how to introduce, maintain, and enforce social distancing at street level and in public places (Vos and Jonas, 2020; Wilder-Smith & Freedman, 2020). Stickle and Felson (2020) address this pandemic as the ‘the largest criminological experiment in history’, and Hodgkinson and Andresen (2020) compare it to other exceptional events, such as natural disasters, big sports events, or terrorist attacks during which the daily routines of thousands of individuals temporarily get altered in ways similar to which the measures taken to reduce the spread of COVID-19 affect the daily routines of millions of individuals. Theory and research suggest this may affect crime in different ways and could result in changes in the spatial patterning of crime. The extant research on COVID-19 and crime, however, remains inconclusive in this regard. Boman and Gallupe (2020), Gerell et al. (2020), Halford et al. (2020), Shayegh and Malpede (2020), Travaini et al. (2020) found that certain crime types decreased during the pandemic, e.g., burglary, property crime, and traffic accidents. However, their findings are not unambiguous; Boman and Gallupe (2020) reported an increase of intimate partner violence (IPV), serious fights, and homicides (Bradbury-Jones & Isham, 2020; Kaukinen, 2020; Piquero et al., 2020). In contrast, Shayegh and Malpede (2020) have neither detected changes in domestic violence nor a reduction in the absolute number of homicides (see also Travaini et al., 2020). Moreover, Shayegh and Malpede (2020) reported a 40% drop in crime, while other studies show no significant crime drop (Ashby, 2020b; Hodgkinson & Andresen, 2020). Ashby (2020a) analysed how calls for service—instead of crime rates—changed during the early weeks of the pandemic in ten large US cities. He focused on eighteen types of incidents, but there were no discernible differences between the period before the first COVID-19 case in the USA and the weeks after (Ashby, 2020a).

Routine Activity Theory (RAT) is harnessed to understand how the COVID-19 containment measures might affect crime in different ways (Ashby, 2020a, 2020b; Buchanan et al. 2020; Felson et al., 2020; Halford et al., 2020). RAT predicts that for a crime to occur motivated offenders and suitable targets must converge in time and space in the absence of capable guardians (Felson et al., 2020). The measures taken to keep the COVID-19 pandemic under control have dramatically altered the routine activities of people, which led to changes in crime rates in several cities around the world (Boman & Gallupe, 2020; Felson et al., 2020; Halford et al., 2020; Hawdon et al. 2020; Stickle & Felson, 2020). The closing of schools, non-essential shops, and leisure activities, as well as the obligation to work from home drastically changed daily mobility patterns. Trips that used to be daily routine, e.g., traveling to school or work, were suddenly prohibited, altering the conditions which impact the convergence of motivated offenders and suitable targets in time and space. RAT projects that lockdown measures will affect in incidents in different ways with some incidents at some places decreasing as a result of the strict measures, e.g., theft in large shopping centres because they had to close or traffic accidents at previously busy intersections, and other incidents at other places increasing as a result of those same measures, e.g., burglaries in abandoned shopping streets (Felson et al., 2020; Shayegh & Malpede, 2020). This may affect the spatial pattern of emergency calls (Felson et al., 2020; Gerell et al., 2020). Consequently, this leads to the hypothesis that: Strict lockdown measures change the spatial pattern of calls for emergency, further referred to as police demand.

Existing research has not considered whether and to what extent this demand for police resources changed and how its spatial pattern was affected by the COVID-19 containment measures. In addition, although we are facing a pandemic, existing research is mainly North America oriented. Ashby (2020b), for example, analysed how crime rates changed during the early months of the COVID-19 pandemic in sixteen US cities, and Hodgkinson and Andresen (2020) and Mohler et al. (2020) observed the crime data of Vancouver, and Los Angeles and Indianapolis respectively. Shayegh and Malpede (2020), who focused on San Francisco and Oakland, identified the need to conduct similar research for European cities. In this study, we address whether and how police demand was affected by COVID-19 lockdown measures and introduce an international perspective to the COVID-19 and crime literature by examining this in Antwerp, Belgium. It is common knowledge that incidents are not evenly distributed in time and space (Weisburd, 2015), but in this context the question rises whether and how this spatial pattern is affected by the pandemic. Therefore, we will examine the spatial pattern of emergency call data before, during, and after the first lockdown in Antwerp, by calculating the generalized Gini coefficient and by performing a spatial point pattern test on the emergency call data of the Antwerp Police Department (APD).

This paper is structured as follows. “Data and methods” section describes the emergency call data set for which the generalized Gini coefficient is calculated and on which the proportion differences spatial point pattern test is performed. The results are reported in “Results” section, followed by a discussion in “Discussion” section. In “Conclusion” section, the most important findings are summarized.

## Data and methods

### COVID-19 in the City of Antwerp

We take as case study the city of Antwerp (Flanders, Belgium). Antwerp is the second largest city of Belgium, counting 530 104 inhabitants (2020) spread over an area of 204.5 km^2^ (2592 people/km^2^), and houses one of Europe’s largest seaports. At the beginning of March 2020, the number of COVID-19 cases in Belgium started to rise, pushing the Belgian National Security Council to announce the federal phase of crisis management on March 12^th^. Initial measures took effect on the 14^th^ of March 2020 and involved the closure of hotels, restaurants, and bars, suspension of all educational activities, and non-essential shops had to close during the weekend. Moreover, teleworking was encouraged and sports events and cultural activities were cancelled. Hospitals did not allow visitors and all non-urgent medical procedures were postponed. A few days later, starting from March 18^th^, stricter regulations were enacted, ultimately resulting in a nationwide lockdown. Until May 4^th^, only essential movements were allowed, teleworking was mandatory (if possible), all non-essential stores were closed, and socializing with anyone else other than your household or one friend at a time (outdoors) was prohibited (Federal Public Service Internal Affairs, 2020).

#### Emergency call data

**Fig. 1:**
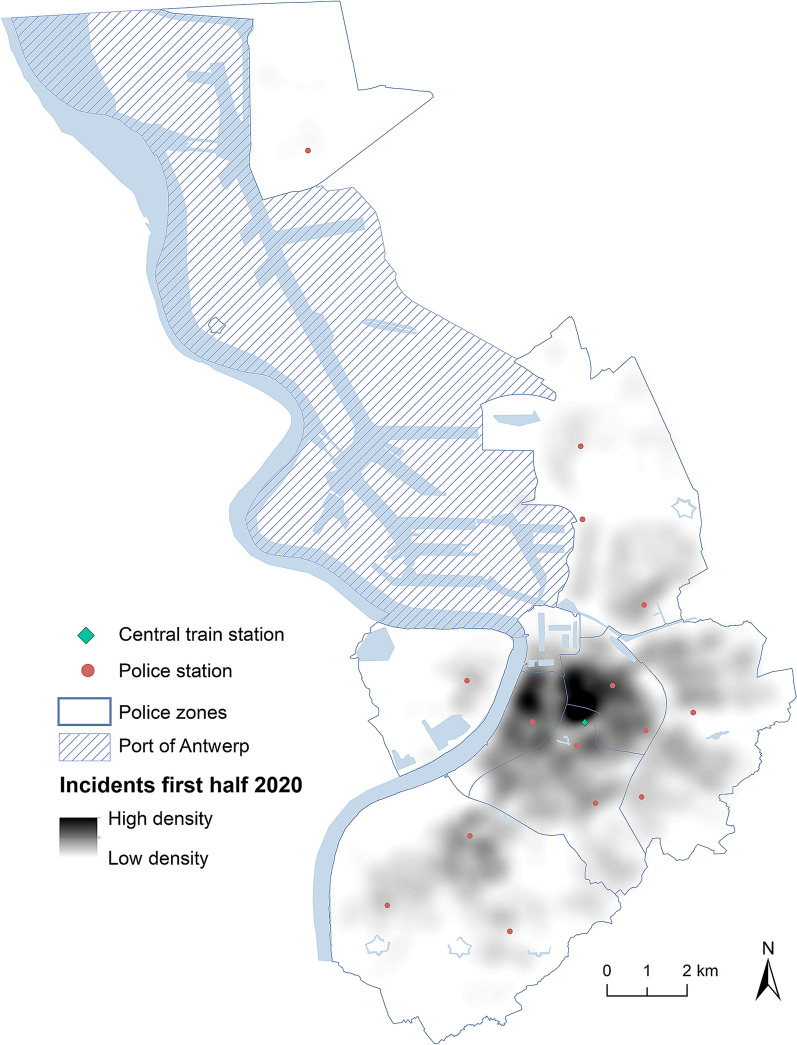
Heat map of the emergency incidents in the first half of 2020 in Antwerp with selected points of interests

The Antwerp Police Department (APD), the largest local police force in Belgium, records data from calls for emergency intervention (pertaining to the police). These emergency calls are handled by the emergency response unit of the Antwerp Police. The city of Antwerp is divided in six police zones, as depicted in Fig. 1. As a rule, the port area is not policed by the APD, although they infrequently respond to calls for emergency intervention within this area. The number of cars of the police emergency unit for each zone depends on, e.g., day/night, week/weekend, and events, but on an average shift approximately 40 intervention vehicles are deployed across the six zones. At night, this number slightly drops.

Emergency call data are the most faithful and extensive source of citizen-reported crimes (Sherman et al., 1989) and are valid indicators for police demand (Davies & Bowers, 2020). They can be used to analyse the presence of the police in time and space, and to predict where police should be present in order to deter crime or at least to decrease response time (Chohlas-Wood et al., 2015; Naoum-Sawaya & Elhedhli, 2013; Rummens et al., 2017). Thus, in this paper emergency call data act as the demand for police service in time and space. In order to examine spatial patterns of police interventions in Antwerp, only emergencies for which a police unit arrived on scene are extracted from the computer aided dispatch system and included in the data set. These interventions are clustered based on their unique incident number, meaning that the sum was taken of the number of patrol units responding to the same incident. In the first half of 2020 (January until June), for example, on average 1.34 (SD: 0.98) patrol units were dispatched to the same incident. The data set contains information on the: (1) priority code of an incident, (2) the date and time of the interventions, (3) type of incident, and (4) place of the incident. The priority codes range from zero to four, with zero being highly urgent, life-threatening incidents, requiring immediate intervention. In this case, officers are allowed to use the vehicle’s siren and/or flashing lights. The urgency is inversely proportional with the priority codes, with code four indicating very low urgency, e.g., graffiti spraying incidents. The date and time are necessary to define the different phases of the COVID-19 measures, and more generally to analyse spatiotemporal patterns of police demand. The data set ranges from January 2019 to June 2020. All incidents were geocoded, either by the researchers (96.1%) or by the Antwerp police (3.9%). In total, 145 167 incidents are included for analysis.

#### Spatial units of analysis

Since police patrol street segments, these are the appropriate spatial unit of analysis. With this underlying focus on police patrol, in total 31 156 street segments, were extracted from the Flemish Road Registry.^1^ A street segment is the section of a street between two intersections, and can be, e.g., a part of a highway or an unpaved road in a park as well (Davies & Johnson, 2015). We have deliberately chosen to include all the different types of roads in the data set, because they are all part of the public domain; the working area of the police. Moreover, by focusing on such a small unit of analysis, we are able to minimize aggregation bias (Andresen & Malleson, 2013; Vandeviver & Steenbeek, 2017). The mean length of the 31 156 street segments is 93.30 m (SD = 159.78).

### Method

The different periods, briefly listed in Table 1, will serve as the periods under analysis throughout this paper. The incidents of 2019 were divided into comparison periods based on the same days and months as in Table 1. During the first period (0), i.e., before the first measures, routine activities had not yet been disrupted and normal life took its course. The two lockdown phases were rather strict, followed by three exit phases, during which the strict rules became systematically less stringent.

**Table 1:** The different phases under analysis

Name	Period	Abbreviation	# days
Period < first measures	2020/01/01–2020/03/13	0	73
Lockdown 1	2020/03/14–2020/03/17	L1	4
Lockdown 2	2020/03/18–2020/05/03	L2	47
Exit phase 1 *Reopening industries, B2B-services and all shops*	2020/05/04–2020/05/17	E1	14
Exit phase 2 *Reopening schools, museums, and animal parks, outdoor sports activities allowed (max. 20 p)*	2020/05/18–2020/06/07	E2	21
Exit phase 3 *Reopening hotels, bars, and restaurants, extending social contact (max. 10 p)*	2020/06/08–2020/06/30	E3	23

To determine the degree of spatial concentration of emergency incidents, we compute the (*generalized*) Gini coefficient. This coefficient is a commonly used statistical measure of the inequality in a distribution, it ranges from 0 (perfect equality) to 1 (complete inequality) (Bernasco and Steenbeek 2017; Maio and Fernando 2007). In the current context, 0 corresponds to a uniformly distributed point pattern over all street segments, while 1 indicates perfect concentration, i.e., all the incidents are concentrated within one single street segment. The value of this coefficient indicates to what extent interventions are spatially clustered and allows to compare the degree of clustering under normal circumstances versus their pattern during the COVID-19 pandemic. Computing the *generalized* Gini coefficient allows us to account for having fewer events than areal units and avoid overestimating the level of concentration in our data (Bernasco and Steenbeek 2017; Vandeviver & Steenbeek, 2017).

A downside to using the Gini coefficient is that it scales with the number of incidents (Mohler et al., 2019). Since longer observation windows are likely to have more incidents and the six periods under study have different lengths (min: 4 days, max: 73 days), direct comparisons between the Gini coefficients of each period are unlikely to be meaningful. To measure the statistical relationship between the number of days and the number of incidents per phase, we compute the Pearson’s correlation coefficient (R) (see Tables 1 and 2). A 0 indicates that no relationship exists, while ± 1 indicates a perfect positive (+ 1) or negative (-1) relationship (Akoglu, 2018). If both variables are correlated, direct comparisons between the Gini coefficients of the phases are not in order. A way around this is standardizing the length of all periods to comparable lengths and then estimating generalized Gini coefficients for each standardized period. We use the shortest time period, i.e. phase lockdown 1 (L1) which consists of four days, to estimate 999 random combinations of four-day periods for each phase longer than four days. For each phase, the average generalized Gini coefficients are then calculated based on the generalized Gini coefficients of each of the 999 random combinations for that phase.

**Table 2:** Absolute and relative number of unique incidents per phase for 2019* and 2020*

	0	L1	L2	E1	E2	E3	Total
2019	18 810 38.88%	1128 2.33%	12 177 25.17%	3916 8.09%	5714 11.81%	6639 13.72%	48 384 100%
2020	19 177 40.22%	1001 2.1%	11 317 23.73%	3619 7.59%	5715 11.99%	6853 14.37%	47 682 100%

*With 2019 and 2020 we only refer to the period corresponding to the different phases

To evaluate the spatial stability of emergency interventions over the different phases, we perform the spatial point pattern test (SPPT) (Andresen, 2009; Wheeler et al., 2018). This area-based nonparametric test can identify the similarity of two spatial point patterns for one phenomenon over time or between several phenomena. The SPPT consists of two levels of analysis: a local and a global level. The global index of similarity, ranging from 0 (no similarity) to 1 (perfect similarity), quantifies the degree of similarity of two point patterns for a given spatial unit of analysis, i.e., the proportion of spatial units exhibiting similar spatial patterns. An exact method to identify a threshold for the global similarity index (S-index) does not exist, but Andresen (2016) suggested a rule of thumb: two spatial point patterns can be considered to approach similarity, if the global S-index ≥ 0.80. The local similarity index calculates the similarity for each areal unit in the analysis and can be easily mapped. For this local index the proportion of incidents can be similar (0) in both data sets, or lower (1) or higher (-1) in the base data than in the test data. It is important to note that global spatial analyses can mask local spatial patterns and, therefore, it is recommended to perform both forms of analysis (Andresen, 2009, 2016).

The original method of the SPPT has two important limitations: (1) the arbitrary choice of a base and test data set, (2) the influence of zero-event street segments (areal units in general) (for a discussion, see Boivin et al., (2019) and Wheeler et al. (2018)). The sample size of the test data will have an influence on its confidence interval and, consequently, on the outcome of the test, which can result in a lower global similarity index if the test set is much larger or in more similar patterns if the test set is smaller than the base set. For areas without incidents in the test data set, the confidence interval will always range from 0 to 0% instead of being small but not zero (Wang, 2013; Wheeler et al., 2018). Therefore, we apply a modified version of the original SPPT, which measures the differences in proportions in each area (Wheeler et al., 2018). Further, because incidents are likely concentrated on a limited number of street segments, most street segments will have zero incidents, and the number of street segments will outnumber the number of emergency calls. To make sure this does not inflate the similarity of two spatial patterns, we calculate the *robust* S-index. This index only considers the number of areal units that have incidents occurring within either the base or test data sets, or in other words, where the events actually occur (Andresen, 2009, 2016). For the proportion test, the Chi-Square approach with Yate’s N-1 continuity correction is applied (set by default in the R function sppt_diff()^2^) (Steenbeek et al., 2020; Wheeler et al., 2018). All calculations are executed in R (version 4.0.1).

## Results

### Spatial concentration of emergency calls in Antwerp

In spite of the extent of the measures taken in Antwerp, the first half of 2020 saw just 702 fewer incidents compared to the first half of 2019 and incidents are similarly distributed over all phases in both years (Table 2). If we look at peak demand, these are the street segments with the highest number of incidents given a certain period, the ten hottest street segments of 2020 generated more incidents than the top ten of 2019, indicating a higher concentration of incidents on these segments. This difference in the number of incidents is levelled out from the ninth segment: for both years the police had to intervene 115 times on exactly the same street segment. The one with the most incidents is for both years located near the central train station of Antwerp and contains 260 and 316 incidents for 2019 and 2020, respectively.

The types of incidents during the strictest phase, lockdown 2 (L2), are qualitatively different from those during the same phase in 2019. Table 3 compares the top ten incident types during phase L2 for 2020 and 2019. In 2020 the police prioritized reacting to public gatherings since these were banned during L2. Later on, it only ranked eighth in Exit Phase 1 (E1). This measure was slightly eased during the exit stage, but large gatherings were not allowed. In 2019 it never appeared in the top ten. In total, for the first half of 2019 only ten incidents were related to interventions regarding social gatherings, in contrast to 903 incidents in the first six months of 2020. Since mid-March 2020 people had to reduce their social contacts to a minimum to slow down the spread of the virus. Consequently, the police were deployed to enforce these measures, in public, but in the case of so called ‘lockdown parties’, also in private places. Furthermore, ‘difficulties in a family with violence’ is not new in the top ten, but in 2019 it never ranked on 3^rd^ place. In 2019, it was not ranked higher than 9^th^ place. The number of burglaries dropped during L2 from 415 in 2019 to 329 in 2020. This may be a result of the stay-at-home order.

**Table 3:** Top ten incident types in the emergency call data set, 2019 and 2020 – phase L2

	**2019**		**2020**	
Ranking	Incident type	Freq	Incident type	Freq
1	Parking violation	998	Gathering	632
2	Entrance/exit obstruction	733	Difficulties with a person	546
3	Difficulties with a person	673	Difficulties in a family with violence	438
4	Traffic nuisance in general	649	Suspicious condition	433
5	Obstructing vehicle	500	Obstructing vehicle	426
6	Burglary	415	Control – on demand	416
7	Suspicious condition	381	Noise nuisance at night—persons	391
8	Traffic accident, material damage with hit and run	362	Traffic nuisance in general	336
9	Brawl—deliberate assault	352	Burglary	329
10	Difficulties in a family with violence	331	Parking violation	316
Total		5394		4263

#### Is police demand concentrated?

The generalized Gini coefficients for the different phases, presented in Table 4, are practically the same for both years, but differ for the different periods. Unlike the overall coefficient for 2020 (0.91), the values in Table 4 are all lower, ranging from 0.36 for the first phase of the lockdown to a coefficient of 0.88 before the introduction of the first measures. This would indicate that the incidents are less concentrated during the separate phases. Put differently, for L1, 1 001 incidents are spread over a total of 787 different street segments, i.e., minimum 79 percent of the incidents happen on diverse street segments. Comparatively, during L2 only 33 percent of the total number of incidents for this period (n = 11,317) took place on a distinct street segment (n = 3775). Although the incidents occur solely on 2.5 percent of the street segments during L1, compared to, for example, 12 percent for L2, the generalized Gini coefficients indicate a more unevenly distributed (hence more concentrated) pattern for L2. Long story short, we observe that as the number of incidents increases, the number of segments on which incidents occurred and the average number of incidents per segment also increase. Thus, the spatial concentration of the incidents is directly proportional to the number of incidents, indicated by a higher Gini coefficient. Because the number of incidents is positively correlated with the length of time period (R = 0.9962, p < 0.001) and each time period has a different length, it is not meaningful to make direct comparisons of Gini coefficients of the different phases (Table 4, columns A and B). Therefore, the average Gini coefficients of random samples of four-day periods for each phase (2020) are calculated and shown in Table 4 (column C). The Gini coefficients computed in this way only slightly differ from 0.36 (L1), and suggest that levels of concentration of police demand are limited and remained mostly unchanged during the entire study period. The coefficient drops very slightly after L1 (from 0.36 to 0.34) and gradually increases as the measures become less stringent.

**Table 4:** Generalized Gini coefficients of the emergency interventions (A) for the different phases of 2019, (B) for the different phases of 2020, and (C) for a random sample of four-day periods for each phase of 2020

	(A) Gen. Gini coefficients 2019	(B) Gen. Gini coefficients 2020	(C) Gen. Gini coefficient 2020 – 4-day periods
0	0.87	0.88	0.36
L1	0.37	0.36	0.36
L2	0.81	0.82	0.34
E1	0.62	0.62	0.34
E2	0.69	0.71	0.35
E3	0.72	0.75	0.38

### Did the spatial pattern of police demand change during the first lockdown in Antwerp?

All global similarity indices in Table 5 approach unity (1) indicating a very high degree of similarity between all phases of the measures, meaning that the spatial patterns of the incidents have not significantly changed during and after this lockdown compared to the first two and a half months of 2020 (period 0). In other words, there are many areal units with a similar spatial pattern in the first six months of 2020. Consequently, the local S-indices are, for each comparison of two phases, almost all equal to 0 indicating a similar proportion of incidents in both phases. For a negligible number of street segments the local S-indices were equal to 1, i.e., segments with a lower proportion of incidents in one of the two phases.

**Table 5:** Bivariate robust global similarity indices for non-zero event street segments (2020)

	0	L1	L2	E1	E2	E3
0		0.9783	0.9998	0.9989	0.9991	0.9991
L1			0.9944	1	0.9993	0.9981
L2				1	1	0.9998
E1					1	1
E2						1
E3						

**Fig. 2:**
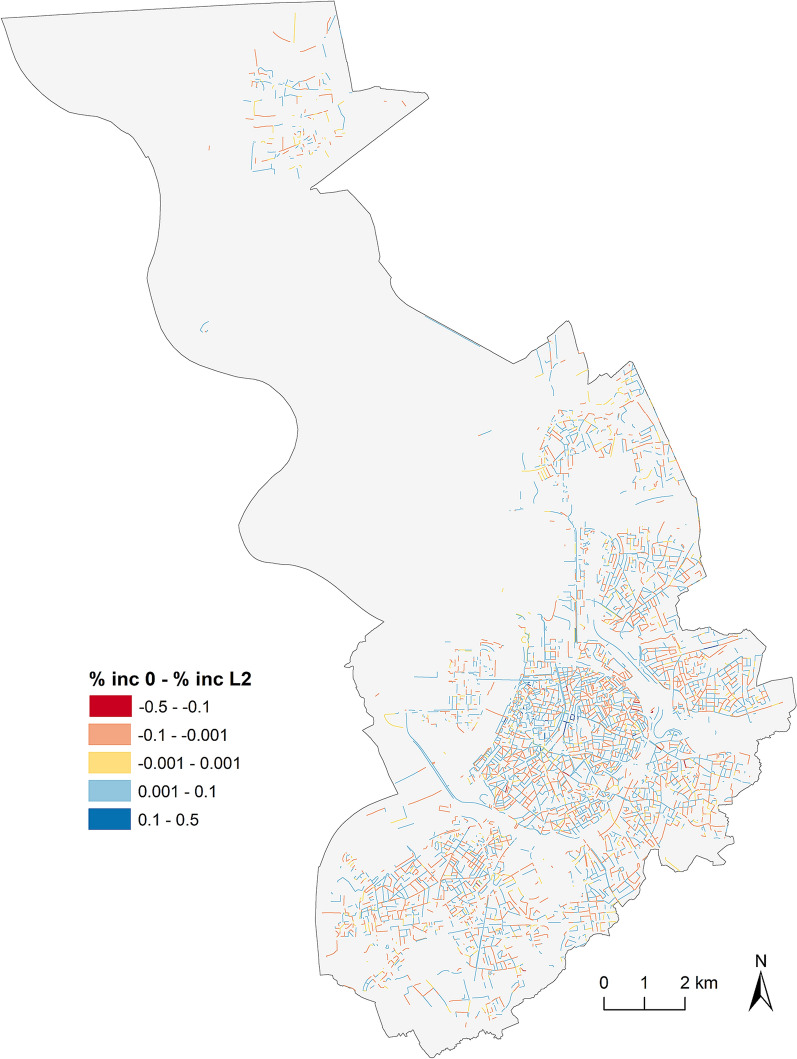
Proportion differences phase 0 and phase L2, 2020

Figure 2 depicts the differences in proportions for all street segments between phase 0 and phase L2 in 2020. Red street segments experienced proportionally more interventions in the second phase of this lockdown, than in phase 0, while blue indicates the opposite, and grey segments experienced the same proportion of incidents in both phases. This Figure demonstrates the absence of major differences in proportions for phases 0 and L2.

Besides the mutual comparison of the six phases in 2020, we ran the SPPT as well to compare the spatial patterns of the same time periods for 2019 and 2020, as shown in Table 6. The outcomes are in accordance to the findings described in the previous paragraphs. Except for the spatial patterns of the periods corresponding to phases 0, all the global S-indices are equal to 1. Thus, the local similarity indices exhibit similarity for almost all street segments, meaning that only minimal differences occur between the spatial patterns of the intervention data of 2019 and 2020. In other words, the strict measures did not change the spatial patterns of police demand in meaningful ways. Last, we performed the differences in proportions test on the most urgent incidents (priority codes 0 and 1). In line with the previous results of the SPPT, all the global S-indices are equal to 1, indicating perfect similarity of the differences in proportions of the two incident patterns.

**Table 6:** Bivariate robust global S-indices for all non-zero street segments, a mutual comparison of the phases in 2019 and 2020

2019 vs 2020	S-index
0	0.9995
L1	1
L2	1
E1	1
E2	1
E3	1

#### Critical reflection on the ‘perfect similar' outcomes of the spatial point pattern test

Although, or maybe just because the very high values of the global similarity indices seem quite exceptional, we should question them. Is it really realistic to assume that the tested spatial patterns are almost identical or how should we interpret ‘perfect similarity’? We are convinced that the SPPT generates a high degree of similarity for a reason, but at the same time we are cautious and expect the high outcomes to be an overestimation of the actual similarity (Boivin et al., 2019; Wheeler et al., 2018). For example, if we compare both periods in Fig. 2, it appears that on 827 street segments with zero events in phase 0, at least one incident occurs in phase L2, with a maximum of 9 incidents for some segments. This indicates that the spatial patterns of both phases are, contrary to what the global S-index tells us, not exactly or ‘perfectly’ the same, and show possible variability that is not included in the global similarity index. Apparently, quite strong differences between spatial units are needed to find significant differences, i.e., even with some fluctuation in base and test points most spatial units are similar, and according to Andresen and Malleson (2011) two spatial patterns become more similar as the spatial unit of analysis becomes smaller, resulting in relatively high S-indices. Consequently, in our analysis ‘perfect’ similarity does not mean that both patterns are exactly the same. The patterns show strong degrees of similarity, but differences between the patterns exist.

## Discussion

The COVID-19 pandemic forced people to adapt their routine activities overnight. Since the first lockdown in March 2020 police are charged with supplementary tasks such as maintaining social distancing, enacting lockdown measures, and enforcing travel bans, in addition to their day-to-day tasks. Laufs and Waseem (2020) conducted a systematic review on the best practices for policing in pandemics. They conclude with the recommendation to shift from a reactive response to the first shock, to the development of a proactive planning strategy for similar events in the future. According to Ashby (2020a) this pandemic had a ‘slow onset’, i.e., in the first weeks no discernible differences were observed in the calls for service. Therefore, it seems likely that police can use the early weeks of any future pandemic to refine and implement contingency plans (Ashby, 2020a). Our results demonstrate that the spatial patterns of emergency incidents in Antwerp show a degree of similarity before, during, and after the lockdown.

In line with most of the research on crime or 911 calls and COVID-19, and as we briefly addressed as well, the types of incidents changed during this lockdown and new types of incidents emerged; violations of the COVID-19 measures. Although analysing the incident types was not the main objective of this paper, our findings give an indication of the impact of the COVID-19 measures on this qualitative aspect. First, during this lockdown the incidents directly resulting from the measures increased. It is difficult to know exactly how many incidents (i.e. which types of incidents) result directly from lockdown measures, but the incidents related to (social) gatherings clearly increased. It would be interesting to subtract all the COVID-19 related incidents from the total number of incidents to get an idea of the actual decrease in incidents during a lockdown. Then the question arises if this would impact the spatial pattern. Second, based on its ranking order, intimate partner violence seems to have increased during this lockdown, which corresponds to the findings of, e.g., Boman and Gallupe (2020). Third, the opposite seems to be true for burglary, which corresponds to the conclusions of Halford et al. (2020), Shayegh and Malpede (2020), and Travaini et al. (2020). A thorough analysis is out of the scope of this paper, but will definitely lead to very interesting insights in potential changes in incident types during a lockdown.

With this analysis we ran into the problem that in the case of our data set the length of a phase is positively correlated with the number of incidents per phase, making direct comparisons of the Gini coefficients of the different phases not meaningful. However, it is valid to state that as the number of incidents increases, the Gini coefficients increase as well. Thus, over a longer period of time, police demand occurs increasingly concentrated, than over a short period of time. To overcome the comparison problem, we calculated for each phase of 2020 the average Gini coefficient of a random sample of four-day periods in that phase. The coefficients are all approximately equal to 0.36, the Gini coefficient of L1, but first slightly drop, followed by a small increase over the exit phases (to 0.38).

The spatial point pattern test is applied because of its attractive properties: it is a convenient and conceptually simple test, which was specifically developed to analyse the degree of similarity of two spatial point patterns. The test is repeatedly applied in criminological research, leading to interesting conclusions about crime patterns (Andresen, 2009, 2016; Andresen et al., 2017; Hodgkinson et al., 2016; Vandeviver & Steenbeek, 2017). The test has proven its value in this paper but, just like the Gini coefficient, also leads to some caution. Perfect similarity does not mean that two point patterns are exactly the same and to find significant differences between two point patterns, strong differences between the spatial units of analysis must be present. Despite its weaknesses, our analysis finds that we must reject the hypothesis that strict lockdown measures change the spatial pattern of police demand. Although there may be a qualitative shift in the type of incidents police responded to, our results indicate that where police were demanded before, during, and after the lockdown in 2020 did not substantially shift. This means that, as stated in Ashby (2020a), the Antwerp Police should not drastically change their patrolling strategy in the first days or weeks of an event with a similar impact as the COVID-19 pandemic. Instead, analysing, e.g., emergency call data, which is increasingly available in police departments, can expose changes on smaller scales, for example, for a specific type of incident or for a specific geographic area. Adapting to these small changes turns out to be more advisable, than drastically adjusting the entire strategy without making sure this is necessary. In the case of Antwerp, detailed data are available on a small scale of analysis, facilitating the implementation of focused crime prevention initiatives, avoiding a potential waste of scarce police resources.

As with all research, this paper is not without its limitations. First, in contrast to, for example, the open crime data policy in the USA, the Antwerp local police only provided us with emergency intervention data of 2019 and 2020 at the time of analysis. Therefore, we could only compare the data of 2020 with comparison periods in 2019. Ashby (2020a), for example, used data from 2016 onwards to forecast the number and types of calls for service during the early weeks of the pandemic. Second, the excessive focus of extant research on the impact of COVID-19 on crime or incident rates, instead of the impact on the spatial pattern of, for example, emergency incidents, makes it difficult to compare our results with existing research. Third, the generalized Gini coefficient, which is specifically developed as a standard methodology to analyse the concentration of crime in the case that spatial units of analysis outnumber incidents (Bernasco & Steenbeek, 2017), still suffers from estimation bias (Mohler et al., 2019). When crime or incident counts are significantly lower than the number of spatial units, estimates of the degree of crime concentration are biased. Therefore, the Gini coefficients in Table 4 may still (slightly) be biased as a result of the low numbers of incidents in each phase (we can expect these low values to be an underestimation of the underlying spatial pattern). This contributes to our awareness that the results should be interpreted and compared with caution (Mohler et al., 2019).

Within this paper we have focused on the first lockdown in Antwerp. Since most countries in Europe, including Belgium, and thus Antwerp, are faced with a second or even third lockdown since the fall of 2020, this will offer an opportunity to compare the spatial patterns of police interventions during both lockdowns. The second lockdown is characterized by strict curfews and assembly and travel bans that are actively enforced. This begs the question how this affects the spatial patterns of police demand? Perhaps seasonality plays out in the police demand patterns during both lockdowns (spring versus fall/winter). All ideas for future research.

## Conclusion

The COVID-19 pandemic acts as a rare natural experiment. With this paper our objective is to add knowledge to the spatial distribution of emergency incidents in extraordinary circumstances, by analysing if changes in people’s daily activities due to strict lockdown measures have the potential to impact spatial patterns of emergency calls in Antwerp. The, at first sight, very unambiguous results of our analysis require some nuance. The SPPT generated S-indices (almost) equal to perfect similarity, meaning that the spatial point pattern of the emergency call incidents has not (significantly) changed before, during or after the first lockdown in Antwerp. However, we argue that smaller local changes are masked by the global S-indices, resulting in an overestimation of the similarity of the point patterns. Another finding that needs further analysis relates to the types of incidents. Lockdown related incidents seem to have increased, just like intimate partner violence. The number of burglaries appears to have decreased during this lockdown. Although the SPPT does not indicate that the measures resulting from the COVID-19 pandemic have caused a significant change in the spatial point pattern of the emergency calls, a substantive change in their daily work, may have had an effect on the daily operation of the Antwerp police force during this lockdown or any similar situation in the future. In such situations, an ongoing analysis of police demand will contribute to avoiding a potential waste of police resources, which is vital in times of crisis.

## Data Availability

Data used in this paper stem from the Antwerp Police Department (APD), are confidential and treated as such. Data sharing requires explicit consent from APD.

## Abbreviations

APD: Antwerp Police Department
SPPT: Spatial point pattern test
S-index: Similarity index
RAT: Routine Activity Theory

## Footnotes

1 Informatie Vlaanderen. (2020). Wegenregister (06/04/2020) [Data file]. Retrieved from: https://download.vlaanderen.be/Producten/Detail?id=6367&title=Wegenregister_06_04_2020#

2 Steenbeek, W.; Vandeviver, C.; Andresen, M.; Malleson, N. (2017). sppt: Spatial Point Pattern Test. R package version 0.1.4. URL: https://github.com/wsteenbeek/sppt
